# Emergence of endothelial subtypes and role of cell cycle control in arterial-venous specification during embryonic vascular development

**DOI:** 10.1016/j.celrep.2025.116368

**Published:** 2025-10-07

**Authors:** Jordon W. Aragon, Elizabeth A. Nelson, Nicholas W. Chavkin, Madeline G. Jackson, Won Heo, Shelby R. Cain, Zaneta Markowska, Grace E. Bradecamp, Gael Genet, Aleksandra Ćwiek, Karen K. Hirschi

**Affiliations:** 1 Department of Cell Biology and Developmental Genomics Center, University of Virginia School of Medicine, Charlottesville, VA 22908, USA; 2 Robert M. Berne Cardiovascular Research Center, University of Virginia School of Medicine, Charlottesville, VA 22908, USA; 3 Center for Developmental Biology and Regenerative Medicine, Seattle Children’s Research Institute, Seattle, WA 98101, USA; 4 Department of Pediatrics, University of Washington School of Medicine, Seattle, WA 98195, USA; 5 Lead contact

## Abstract

During vascular development, endothelial cells (ECs) specify into arterial, capillary, and venous subtypes to form a circulatory network. While the cell cycle state enables postnatal arterial-venous specification in a flow- and tissue-specific manner, its role during embryogenesis remains unclear. To investigate this, we isolated ECs at embryonic day (E)8.0 (pre-flow), E8.5 (post-flow), and E9.5 and performed single-cell RNA sequencing. Arterial, venous, and hemogenic subtypes emerged with significant enrichment of cell-cycle-related pathways. Using Fucci embryos, ECs were sorted into early G1, late G1, and S/G2/M states and profiled by bulk RNA sequencing. Integration with our single-cell data showed that venous ECs aligned with early G1 and arterial ECs aligned with late G1 transcriptional profiles, consistent with imaging of Fucci embryos. Deleting cell cycle inhibitor *Cdkn1b* (p27) in embryonic ECs disrupted arterial-venous development, demonstrating that cell cycle control plays a critical role in embryonic arterial-venous specification at the earliest stages of vascular development.

## In brief

Aragon et al. show that arterial and venous endothelial cells are enriched in distinct cell cycle states before the onset of blood flow and that this precise cell cycle control is necessary for the specification of these endothelial subtypes within the developing embryo.

## Graphical Abstract

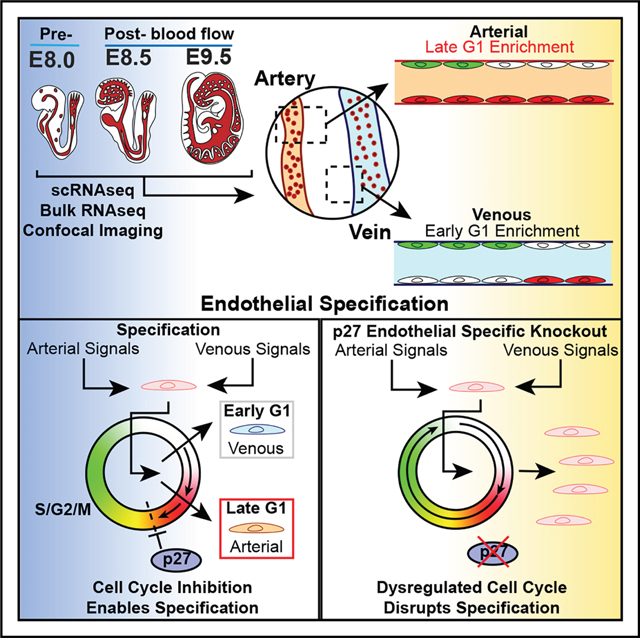

## INTRODUCTION

During blood vascular development, endothelial cells (ECs) arise from mesodermal progenitors and form a primitive capillary network that remodels into arterial, venous, and capillary vessels. Specification of these distinct EC subtypes is essential during development, with loss leading to embryonic lethality.^1–4^ Mechanisms regulating EC specification during the earliest stages of arterial-venous development are not fully defined.

Many studies of arterial-venous specification have focused on postnatal windows of development, using the murine retinal vascularization model.^5^ Our studies using this model and Fucci cell cycle reporter mice revealed that flow-mediated EC cell cycle control plays a critical role in arterial-venous specification during postnatal angiogenesis. Venous- and arterial-specific shear stress promote early G1 vs. late G1 cell cycle states, respectively, enabling BMP4-mediated venous or transforming growth factor β (TGF-β)-mediated arterial gene expression.^5,6^ Importantly, we found that reestablishing EC cell cycle control in hyperproliferative retinal vascular malformations restores endothelial identity, structure, and function.^6^ Other studies also link endothelial cell cycle state and identity during postnatal and tissue-specific angiogenesis.^7–9^

Despite these studies indicating that fluid shear stress upon the endothelium leads to cell-cycle arrest and specification, recent evidence shows that ECs proliferate in high-shear environments,^10,11^ embryonic EC identity emerges before blood flow, and arterial-venous ECs can be generated in stem cell culture systems without fluid shear.^12,13^ Thus, whether EC cell cycle control plays a common role in arterial-venous specification remains an open question. Further elucidating universal mechanisms by which EC identity is established will provide the insights needed to optimize the derivation of distinct EC subtypes from human stem cells, drive arterial-venous formation in tissues with impaired blood flow, and treat prevalent pathologies in which ECs are hyperproliferative and arterial-venous identities are lost.^6^

To address whether cell cycle control is necessary for embryonic vascular development, we defined the emergence of endothelial subtypes during vascular development, beginning prior to the onset of blood flow. We isolated ECs at embryonic day (E)8.0 (0–6 somites, pre-flow), E8.5 (>7 somites, post-flow), and E9.5 and subjected them to single-cell RNA sequencing (scRNA-seq). Gene Ontology (GO) terms associated with cell cycle control were significantly enriched over time, and arterial identity was strongly correlated with growth arrest. Using bulk RNA-seq and imaging of Fucci embryos, we found that arterial ECs were highly enriched in late G1 and venous ECs were highly enriched in early G1. Genetically deleting cell cycle inhibitor p27 in ECs at E7.5–8.5 revealed that loss of endothelial cell cycle control disrupts arterial-venous specification. The insights gained can be applied to tissue engineering and regenerative medicine approaches, and the sequencing datasets are valuable resources for further investigation of vascular development.

## RESULTS

### Single-cell analysis of ECs during early embryonic development

**Figure 1.**
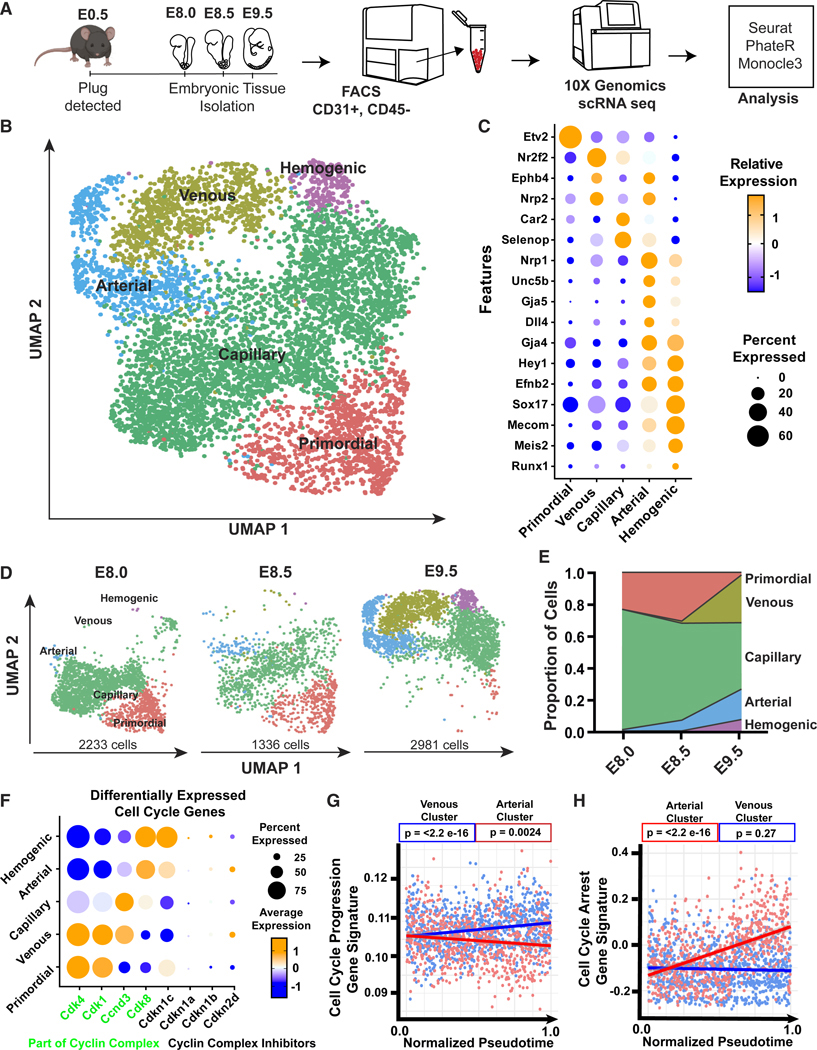
Single-cell analysis of developing embryonic ECs (A) Schematic for collection of embryonic ECs and scRNA-seq. (B) UMAP dimensionality reduction showing all ECs (6,550 cells). (C) Dot plot of genes used to identify endothelial subtypes. (D) UMAP dimensionality plots of different embryonic time points. (E) Fraction of EC subtypes per time point. (F) Differentially expressed cell cycle genes in endothelial subtypes. (G and H) Simple linear regression analysis of cell cycle progression gene signature (G) and cell-cycle arrest gene signature (H) of arterial and venous clusters, as a function of pseudotime.

To investigate transcriptomic profiles of ECs during specification throughout the embryo, we isolated CD31^+^CD45^—^ ECs using fluorescence-activated cell sorting (FACS) from E8.0 (0–6 somites, pre-flow), E8.5 (>7 somites, post-flow), and E9.5 C57BL/6 wild-type embryos and then performed scRNA-seq utilizing the 10× Genomics Chromium pipeline (Figure 1A; Table S1). After processing and filtering out poor-quality cells, we obtained a dataset containing 6,550 ECs. Seurat^14,15^ was used to perform uniform manifold approximation and projection (UMAP) dimensionality reduction and clustering of all time points, and we identified five distinct EC subtypes—primordial, venous, capillary, arterial, and hemogenic (Figure 1B)—which were annotated based on significant expression of known subtype-enriched genes (Figure 1C). Primordial ECs are enriched for *Etv2*, a known master regulator of EC differentiation.^16^ Venous ECs are enriched for *Nr2f2*, together with *Ephb4* and *Nrp2*, and lack expression of other subtype-enriched genes. Capillary ECs are enriched for *Car2*^17^ and *Selenop*, while *Nrp1*, *Unc5b*, *Gja5*, *Dll4*, *Gja4*, *Hey1*, *Efnb2*, and *Sox17* are enriched in the arterial cluster. Hemogenic ECs show enriched expression of *Mecom*, *Meis2*, and *Runx1*, along with some arterial-enriched genes, as expected, given their origin in the aorta-gonad-mesonephros region. GO analysis of the clusters revealed angiogenesis, cell adhesion, morphogenesis, and vascular developmental pathways enriched in venous, capillary, and arterial EC subtypes (Figure S1A).

Over time, EC subtypes shift in proportion as the embryo develops (Figure 1D). At E8.0, 75% of ECs were identified as capillary and 23% as primordial, while venous, arterial, and hemogenic ECs were each <1% of the population. At E8.5, capillary ECs decreased to 61% of the population and primordial to 30%, while arterial increased to 6%; venous and hemogenic remained at ∼1%. At E9.5, capillary and primordial ECs decreased to 41% and 1%, respectively; conversely, arterial increased to 20%, venous to 30%, and hemogenic to 7% (Figure 1E).

To explore EC specification over time, we performed dimensionality reduction with PhateR^18^ and generated pseudotime values of all 6,550 ECs using Monocle3^19^ (Figure S1B). Primordial and a subpopulation of capillary ECs exhibit low pseudotime, as they emerge at the earliest time point, E8.0. Arterial and hemogenic cells exhibit medium-to-high pseudotime, and venous cells exhibit the highest pseudotime, consistent with other studies suggesting that venous ECs develop after arterial ECs.^20^ We used Seurat module scoring to calculate arterial and venous endothelial subtype scores for each EC in the dataset, using the arterial-enriched genes *Efnb2*, *Dll4*, *Hey1*, *Gja4*, *Gja5*, and *Unc5b* and the venous-enriched genes *Nr2f2*, *Nrp2*, and *Ephb4*. Linear regression analysis revealed a significant positive correlation between pseudotime and the arterial or venous module score (Figure S1C), consistent with real-time population changes (Figures 1D and 1E).

To investigate transcriptional differences among embryonic ECs during specification, we performed GO analysis of genes correlated with pseudotime. Many proliferation-related GO terms were significantly enriched over pseudotime, suggesting a key role for cell cycle control as EC subtypes are specified (Figure S1D). Since EC identity is regulated in a cell-cycle-dependent manner during postnatal angiogenesis,^6,7,21–23^ we further investigated the relationships between embryonic EC identity and cell cycle control.

Differential gene expression analysis using genes within the “positive regulation of cell cycle” GO category (GO: 0045787) and “cyclin complex inhibitor” category (GO: 0004861) showed differences in cell cycle gene expression among the different EC subtypes (Figure 1F). To further investigate this relationship, a “cell cycle progression score” was calculated using all genes within GO: 0045787, and a “cell-cycle arrest score” was calculated using all genes in GO: 0004861. The cell cycle progression score significantly decreased in arterial ECs over pseudotime and increased in venous ECs over pseudotime (Figure 1G), suggesting subtype-specific expression of cell cycle regulatory genes. Further analysis revealed that arterial ECs significantly increase in cell-cycle arrest score over pseudotime, consistent with previous findings,^7,21–23^ while venous ECs did not have a significant change in cell-cycle arrest gene enrichment (Figure 1H). These data suggest that there is a transcript-level association between endothelial subtype and cell cycle regulatory genes.

### Differential gene expression in ECs in distinct cell cycle states

**Figure 2.**
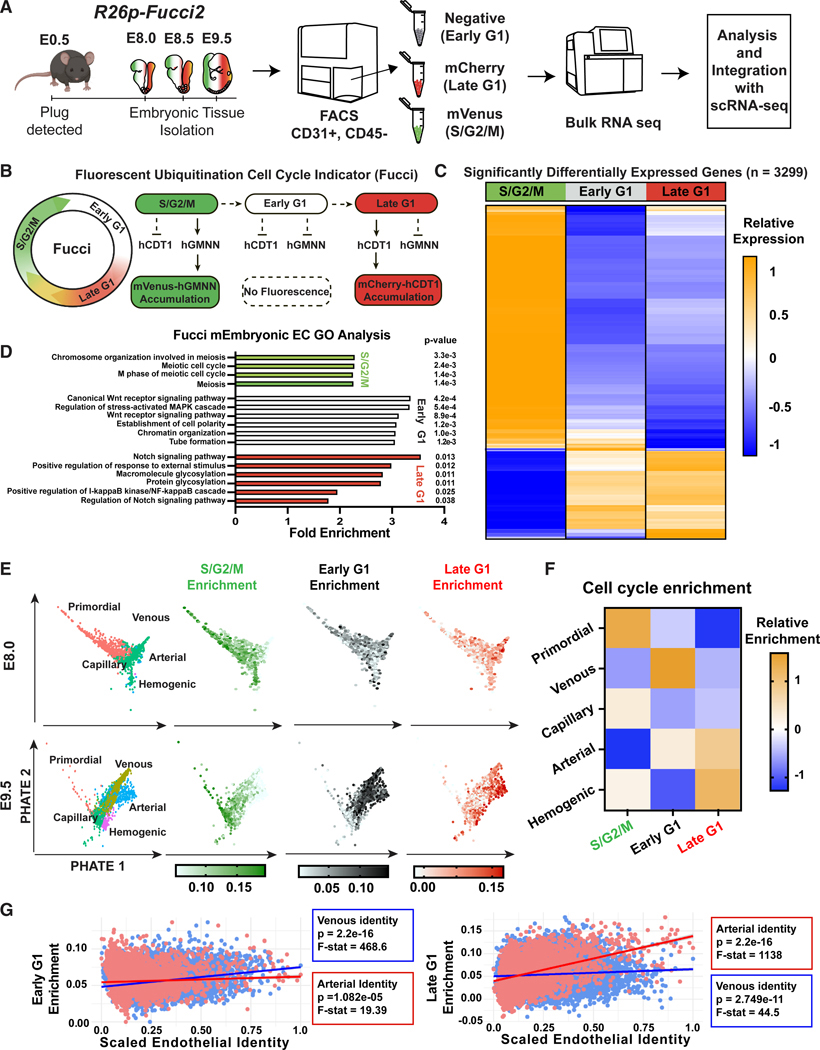
EC identities transcriptionally correspond to different Fucci cell cycle states (A) Schematic of EC isolation from *R26p-Fucci2* embryos at E8.0, E8.5, and E9.5; separation into early G1, late G1, and S/G2/M cell cycle states; and data integration with scRNA-seq data from Figure 1. (B) Schematic of *R26p-Fucci2* reporter mice for detection of cells in distinct cell cycle states. (C) Pairwise analyses showing differentially expressed genes of ECs in different cell cycle states. (D) GO enrichment analysis of differentially expressed genes from ECs in S/G2/M, early G1, and late G1. (E) Enrichment of differentially expressed genes in S/G2/M, early G1, and late G1 in individual ECs plotted in potential of heat diffusion for affinity-based transition embedding (PHATE) dimensionality reduction plot. (F) Heatmap of average enrichment of S/G2/M, early G1, and late G1 in different EC subtypes. (G) Simple linear regression and F-statistical analysis of total ECs plotted as arterial or venous identity vs. late G1 or early G1 gene enrichment.

To better define the relationships between EC identity and cell cycle state, we used *R26p-Fucci2* cell cycle reporter mice. We isolated CD31^+^CD45^−^ ECs from E8.0, E8.5, and E9.5 *R26p-Fucci2* embryos and separated them using FACS into early G1, late G1, and S/G2/M subpopulations based on endogenous *R26p-Fucci2* expression (Figure 2A; Table S2). Cells in early G1 have no reporter fluorescence (both CDT1-mCherry and GEMININ-mVenus fusion proteins are degraded); in late G1, cell nuclei are red (CDT1-mCherry is not degraded); and in S/G2/M, nuclei are green (GEMININ-mVenus is not degraded)^24^ (Figure 2B). We conducted low-input paired-end bulk RNA-seq of ECs in the three distinct cell cycle states at each time point. We used DESeq2 to identify significantly differentially expressed genes within each cell cycle state in all time points combined and found 3,299 differentially expressed genes (Figure 2C), with many cell cycle genes showing differential expression in distinct cell cycle states (Figure S1E).

To gain insight into enriched pathways in each cell cycle state, we performed GO analysis. Proliferation-related GO terms were highly enriched in S/G2/M, as expected (Figure 2D). Early G1 ECs were enriched in Wnt and MAPK signaling, as well as cell polarity and tube formation-related genes, while late G1 ECs were enriched for Notch and nuclear factor κB (NF-κB) signaling and protein glycosylation-related genes. To investigate the relationships between cell cycle state and EC identity, we applied a module score for each cell cycle state, based on differentially expressed genes, to each cell in our scRNA-seq dataset (Figures 2E and S1F). Primordial and capillary EC clusters exhibited enrichment in S/G2/M score, suggesting a high association with proliferation. The venous cluster showed strong enrichment for early G1 score, while arterial ECs showed enrichment for late G1 score, showing a strong association between EC identity and different cell cycle states. Hemogenic ECs were also highly enriched for late G1, consistent with our previous finding that hemogenic specification requires G1 arrest (Figure 2F).^22^ Linear regression analysis comparing EC identity and cell cycle state revealed that all relationships are significant. However, F-statistical analysis indicates that arterial identity and late G1 transcriptional profiles better overcome the noise of the dataset and are more strongly linked; venous and early G1 are similarly better aligned (Figure 2G). These data suggest that during embryonic vascular development, arterial ECs transcriptionally correspond with the late G1 cell cycle state and venous ECs with early G1.

### Endothelial cell cycle states in the developing embryo

To investigate whether ECs in arteries and veins *in vivo* are enriched for different cell cycle states, before and after flow, we performed confocal microscopy of E8.0–10.5 *R26p-Fucci2* embryos. We collected E8.0, E8.5, E9.5, and E10.5 *R26p-Fucci2* embryos, fixed and sectioned them, performed immunostaining with anti-ERG1/2/3 (EC nuclear marker), and imaged them using fluorescence confocal microscopy. To confirm the accuracy of Fucci cell cycle states, we immunostained embryonic sections with anti-phospho-histone H3 (pHH3) and confirmed that all pHH3+ ECs are within S/G2/M, with none detected in early G1 or late G1 (Figure S2A). The dorsal aorta (DA; largest embryonic artery), sinus venosus (SV; precursor venous vessel), and cardinal vein (CV; largest embryonic vein) were identified via a combination of anti-DLL4 and anti-EMCN immunostaining (Figure S2B), morphology, and location; therein, ERG^+^ EC nuclei in different cell cycle states were quantified using ImageJ.

**Figure 3.**
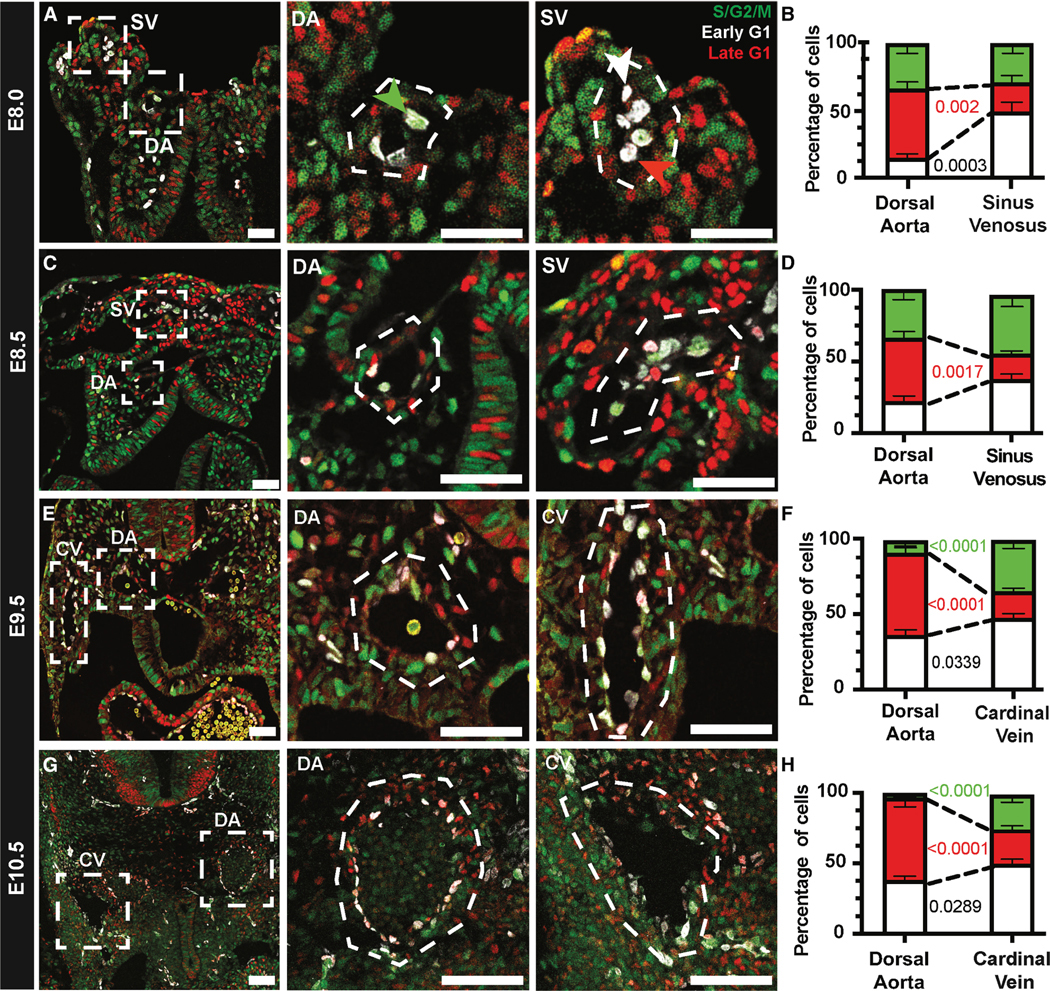
ECs in major arterial and venous vessels reside in distinct cell cycle states (A) Representative confocal images of E8.0 *R26p-Fucci2* embryo sections; scale bar: 140 μm. Magnified images of dorsal aorta (DA) and sinus venosus (SV); scale bars: 40 μm. Green arrow = S/G2/M EC, white = early G1, and red = late G1. (B) Quantified cell cycle states in E8.0 DA and SV (DA *n* = 15 embryos and SV *n* = 7 embryos). (C) Representative confocal images of E8.5 *R26p-Fucci2* embryo sections; scale bar: 150 μm. Magnified images of DA and SV; scale bars: 50 μm. (D) Cell cycle states in E8.5 DA and SV (DA *n* = 17 embryos and SV *n* = 8 embryos). (E) Representative confocal images of E9.5 *R26p-Fucci2* embryo sections; scale bar: 165 μm. Magnified images of DA and cardinal veins (CVs); scale bars: 55 μm. (F) Cell cycle states in E9.5 DA and CV (DA *n* = 24 embryos and CV *n* = 24 embryos). (G) Representative confocal images of E10.5 *R26p-Fucci2* embryo sections; scale bar: 170 μm. Magnified images of DA and CV; scale bars: 55 μm. (H) Cell cycle states in E10.5 DA and CV (DA *n* = 12 embryos and CV *n* = 12 embryos). Standard error of mean (SEM) ±; two-way ANOVA. All images were immunostained with EC nuclear marker anti-ERG1/2/3 to identify ECs in early G1 (ERG^+^, reporter negative), late G1 (ERG^+^ mCherry^+^), and S/G2/M (ERG^+^ mVenus^+^).

At E8.0, ECs in different vessel subtypes were already significantly enriched in different cell cycle states (Figure 3A): DA ECs were significantly enriched in late G1, while SV ECs were significantly enriched in early G1. Both populations also contained a significant and equal proportion of ECs in S/G2/M (Figure 3B). At E8.5 (Figure 3C), DA ECs were significantly enriched in late G1 compared to the SV, and both vessel types continued to have significant ECs in S/G2/M (Figure 3D). At E9.5 (Figure 3E), DA ECs were enriched for late G1, with a significant decrease in S/G2/M ECs compared to the CV; conversely, CV ECs were significantly enriched for early G1 (Figure 3F). At E10.5 (Figure 3G), DA ECs continued to be significantly enriched in late G1 and CV ECs in early G1, with a significantly higher proportion of S/G2/M cells, compared to DA (Figure 3H). We found similar trends in other embryonic vessels; for example, ECs in the intra-carotid artery, the major arterial vessel within the cranial vasculature (Figure S2C), were significantly enriched in late G1 (Figure S2D). Using anti-Dll4 immunostaining to denote arterial (DLL4^High^) and venous (DLL4^Low^) vessels in the E9.5 somitic vasculature (Figure S2E), we found arterial ECs enriched in late G1 and venous ECs enriched in S/G2/M and early G1 (Figure S2F). These studies reveal that ECs in embryonic arteries are significantly enriched in late G1 and ECs in embryonic veins in early G1 during vascular development, this cell-cycle-state difference is present before the onset of systemic blood flow, and EC proliferation (S/G2/M) is reduced across developmental time.

### Endothelial cell cycle control is required for arterial-venous specification

**Figure 4.**
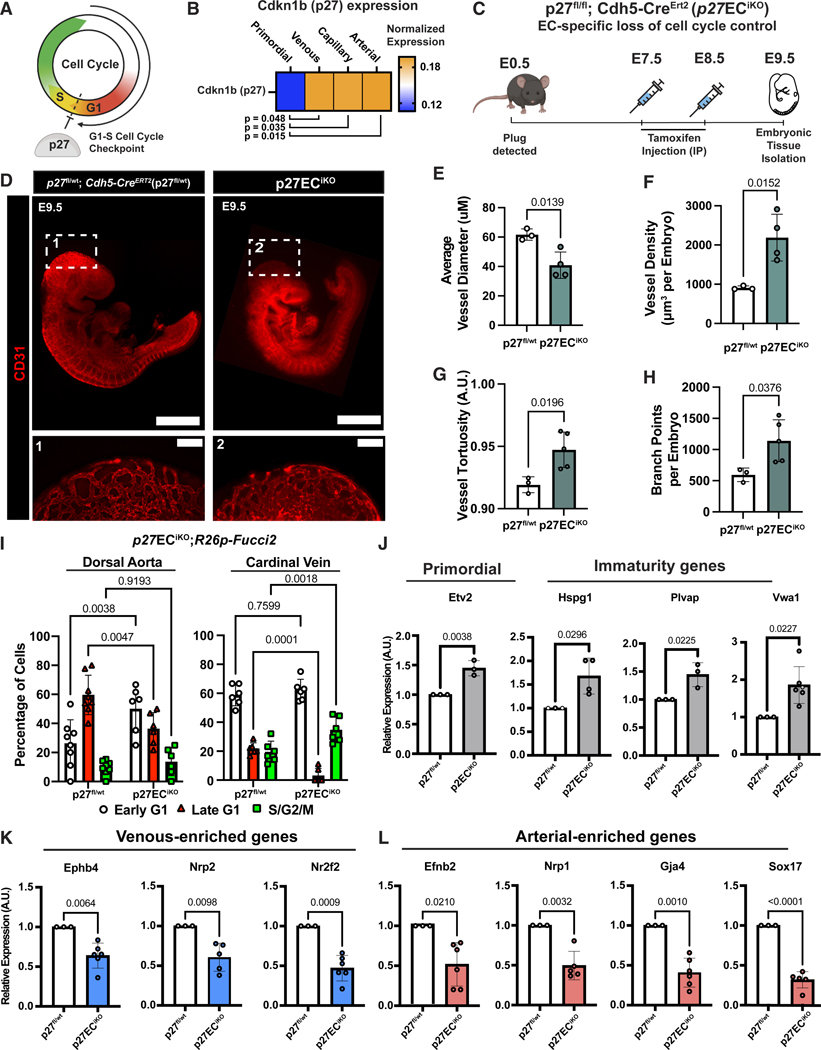
Embryonic endothelial cell cycle control is necessary for arterial and venous specification (A) p27 inhibits cell cycle in late G1 and prevents transition into S phase. (B) *Cdkn1b* (p27) expression in primordial, venous, capillary, and arterial clusters from scRNA-seq data in Figure 1; results are of two-way ANOVA statistical analysis. (C) Schematic of tamoxifen injection of *p27*EC^iKO^ mothers and collection of E9.5 embryos. (D) Representative wide-field images of E9.5 *p27*^*fl/wt*^ control (left, 1) vs. *p27*EC^iKO^ (right, 2) embryos immunostained with anti-CD31; scale bars: 500 μM. Magnified images of cranial vasculature; scale bars: 120 μM and 160 μM. (E–H) Average vessel diameter (E), vessel density (F), vessel tortuosity (G), and vessel branch points (H), calculated using Imaris (*p27*^*fl/wt*^ *n* = 4 and *p27*EC^iKO^ *n* = 7). (I) Quantification of EC cell cycle states in E9.5 DA and CV of *p27*EC^iKO^;*R26p-Fucci2* embryos (control *n* = 8 embryos and *p27*EC^iKO^;*R26p-Fucci2 n* = 6 embryos). (J–L) RT-qPCR of primordial/immaturity, venous-enriched, and arterial-enriched genes in FACS-isolated CD3^+^CD45^−^ ECs (*p27*^*flwtl*^ *n* = 3 and *p27*EC^iKO^ *n* = 3–6) (SEM ±, all unpaired *t* tests, from multiple litters, *p27*EC^iKO^ paired with *p27*^*fl/wt*^ littermate controls). a.u., arbitrary units.

To investigate whether embryonic EC cell cycle control is necessary for specification, we genetically induced EC cell cycle dysregulation at the onset of vascular development. We previously found that cell cycle inhibitor *Cdkn1b* (p27), which inhibits CDK4/6 to prevent G1-S transition and promote late G1 state (Figure 4A),^25^ is also a critical regulator of EC specification during postnatal angiogenesis.^21,26^ Therefore, we evaluated p27 expression within our scRNA-seq dataset and found that p27 is significantly enriched in more specified subtypes (venous, capillary, and arterial ECs) but not primordial ECs (Figure 4B), suggesting that p27 may play a role in subtype specification. To test this, we generated EC-specific inducible p27 deletion mice (*p27*^*fl/fl*^*; Cdh5*-Cre^ERT2^: *p27*EC^iKO^) and induced p27 deletion at E7.5–8.5, after EC differentiation and primitive capillary plexus formation. Embryonic tissues were collected at E9.5 to investigate changes in vascular morphology and specification (Figure 4C); *p27*^*fl/wt*^*; Cdh5-*Cre^ERT2^+ (*p27*^fl/wt^) littermates were used as controls.

Deletion of p27 in ECs was confirmed in *p27*EC^iKO^ embryos by significant reduction of *Cdkn1b* expression in CD31^+^CD45^—^ ECs compared to controls via RT-qPCR (Figure S3A). Using anti-CD31 immunostaining and wide-field microscopy to visualize vascular morphology, we found impaired embryonic growth and vascular development (Figure 4D). Cryo-sectioning and immunostaining for pHH3 in E9.5 ECs revealed significantly higher proliferation in *p27*EC^iKO^ ECs vs. controls (Figure S3B), consistent with loss of cell cycle control. Cryo-sectioning of embryonic tissue and co-immunostaining for CD31, DAPI, and cleaved caspase-3 revealed significantly higher apoptotic ECs in *p27*EC^iKO^ embryos (Figure S3C). Due to reports of Cre toxicity within this *Cdh5*-Cre^ERT2^ line,^27^ we immunostained with anti-CD31 and performed wide-field microscopy of wild-type littermate embryos bearing no Cre but that were similarly treated with tamoxifen (Figure S3D).

To further investigate changes in 3D vascular structure, E9.5 *p27*EC^iKO^ and control embryos were optically cleared using an adapted protocol^28^ and visualized with fluorescence light-sheet microscopy (Figure S3E). Light-sheet images were rendered in 3D and analyzed using Imaris 10.2.0 (Video S1). Quantification of vascular parameters across all embryonic vessels revealed significantly decreased vessel diameters (Figure 4E), significantly higher vessel density (Figure 4F), significantly higher vessel tortuosity (Figure 4G), and significantly increased vessel branch points (Figure 4H) in *p27EC*^iKO^ embryos, consistent with EC hyperproliferation and impaired vascular remodeling.

To further quantify changes in EC cell cycle state in *p27EC*^iKO^ embryos, we generated *p27*EC^iKO^;*R26p-Fucci2* embryos. Using cryo-sectioning and immunostaining, we found that late G1 ECs were significantly reduced in both the DA and CV, as expected. In addition, early G1 ECs were significantly increased in the DA, and S/G2/M ECs were significantly increased in the CV (Figure 4I).

To determine if disrupted cell cycle control, increased EC proliferation, and vascular density were associated with loss of arterial-venous identity, we FACS-isolated CD31^+^CD45^−^ ECs from E9.5 *p27*EC^iKO^ and control embryos, isolated RNA, and measured gene expression using qPCR. We found genes indicative of primordial (*Etv2*) and immature (*Hspg2*, *Plvap*, and *Vwa*, defined in other studies^29,30^) ECs enriched in *p27*EC^iKO^ embryos (Figure 4J). Conversely, venous- (*Ephb4*, *Nrp2*, and *Nr2f2*) (Figure 4K) and arterial- (*Efnb2*, *Nrp1*, *Gja4*, and *Sox17*) enriched genes were significantly decreased in *p27*EC^iKO^ embryos (Figure 4L). When mothers were injected at later time points (Figure S3F), significantly decreased arterial- and venous-enriched gene expression remained consistent (Figures S3G and S3H). Thus, loss of endothelial cell cycle control during embryonic vascular development results in impaired arterial-venous specification.

## DISCUSSION

Our studies reveal that during early vascular development, distinct EC identities are correlated with different cell cycle states beginning at the earliest stages of specification, prior to the onset of blood flow. We found arterial ECs enriched in late G1 and venous ECs enriched in early G1, similar to postnatal vasculature.^21,23^ Furthermore, we found that endothelial cell cycle control is required for proper arterial-venous specification and vascular development. Mechanistically, we found cell cycle inhibitor p27 to be a critical regulator of endothelial cell cycle control, enabling growth arrest, arterial-venous gene expression, and vascular remodeling. These findings are consistent with other developmental studies showing that hemogenic ECs must also undergo growth suppression to become specified.^9,21,23^

A number of recent studies have shown the necessity of cell cycle control for the establishment of EC subtype identity during postnatal and tissue-specific angiogenesis.^6,7,21,31^ Studies, including our own, have identified blood flow as an inducer of endothelial cell cycle control in these contexts.^8,21^ However, it was unclear whether the endothelial cell cycle state is a common or necessary mechanism that governs subtype specification during early vascular development prior to the onset of blood flow. Our study reveals that the endothelial cell cycle state is differentially regulated in embryonic arterial and venous vessels before the onset of flow, suggesting that cell cycle control, as an intrinsic mechanism driving specification, can occur without flow induction.

A better understanding of signaling pathways that regulate early vs. late G1 states in specifying ECs could provide targetable pathways for directing specification. Our bulk RNA-seq data from embryonic ECs in distinct cell cycle states suggest candidates for future studies, including differentially expressed cell cycle regulatory genes and Wnt signaling. Wnt ligands are known to suppress EC proliferation, but it is unclear whether Wnt induction can induce a specific cell cycle state.^32–34^ Enrichment of Wnt activity in early G1 suggests a potential role in promoting cells in early G1 and/or regulating venous specification. Conversely, late G1 ECs are enriched for protein glycosylation pathways, a process that influences hemogenic EC fate determination^35^ and may influence arterial EC specification.

Our findings suggest that cell cycle regulation is critical for EC specification at the earliest stages of vascular development prior to blood flow. Such insights can be applied to human stem cell systems to optimize the generation of EC subtypes and improve the effectiveness of tissue engineering and regenerative medicine applications. Additionally, these studies offer a promising basis for targeting endothelial cell cycle control in many prevalent pathologies where ECs are hyperproliferative and arterial-venous identity is lost, including tumor angiogenesis, pulmonary hypertension, macular degeneration, and diabetic retinopathy.^36–39^ We have previously shown that reestablishment of endothelial cell cycle control prevents or resolves vascular malformations in mouse models of hereditary hemorrhagic telangiectasia.^6^ Repurposing US Food and Drug Administration (FDA)-approved cell cycle modulators may be a viable therapeutic avenue for the treatment of other prevalent vascular diseases.

### Limitations of the study

These studies do not address post-translational control of p27 and other cell cycle regulators. The activity of p27 is controlled primarily through protein modifications, such as phosphorylation and ubiquitination.^40,41^ N-glycome modification of cell cycle regulators, including p27, is also an important regulator of cell cycle control^6^ and could therefore be affecting EC specification. We previously found that p27 is enriched through a laminar-flow-mediated pathway,^26^ but flow-independent mechanisms regulating p27-mediated cell cycle control in ECs remain unknown. Targeted deletion of p27 in ECs reduced venous- and arterial-associated genes, suggesting that p27 may regulate transition into G1 and early-to-late G1 transition. p27 is implicated in the regulation of other cell cycle checkpoints in S, G2, and M phases.^41^ Enrichment of primordial and capillary ECs in S/G2/M suggests that EC differentiation and capillary specification may also be mediated via a cell-cycle-dependent mechanism. It is suggested that G2 is also a critical window for EC fate decisions,^42^ so further investigations using other Fucci systems that distinguish these phases are of interest.

### RESOURCE AVAILABILITY

#### Lead contact

Requests for further information and resources should be directed to and will be fulfilled by the lead contact, Karen K. Hirschi (kkh4yy@virginia.edu).

#### Materials availability

No new reagents were generated in this study. Further information about reagents and requests for resources are available upon request to the authors.

#### Data and code availability

- All data within this publication are available and will be shared upon request to the lead contact. All raw RNA-seq files are deposited within the NCBI GEO and are publicly available as of the date of publication. Accession numbers (GEO: GSE287112 and GEO: GSE287115) are listed in the key resources table.
- All relevant code to this paper is available upon request and will be fulfilled by the lead contact.
- Any additional information required to reanalyze the data reported in this paper is available upon request and will be fulfilled by the lead contact.

## STAR★METHODS

### EXPERIMENTAL MODEL AND STUDY PARTICIPANT DETAILS

All animal experiments were approved by the University of Virginia Animal Use and Care Committee (protocol #4277) and complied with all ethical regulations. All mice were housed at the University of Virginia in a vivarium with a 12-h day and night cycle environment with *ad libitum* availability of chow diet and water. All mouse strains used are C57BL/6 background. Mice were combined in breeding pairs and monitored for presence of a vaginal plug. Upon detection of a plug, this was denoted as embryonic day (E)0.5. For E8.0 and E8.5 embryos, additional counting of somite pairs was performed to accurately separate embryos prior to onset of blood flow (0–6 somite pairs) and post onset of flow (>7 somite pairs). Male and female embryos were included in all studies. Mouse strains used in this study include:*C57BL/6 (Jax: 000664) (wild type),* E8.0, E8.5 and E9.5 *C57BL/6* embryos were used for these studies. *R26p-Fucci2*^24^ (Riken: CDB0203T), to maximize bulk RNA sequencing efficiency, *R26p-Fucci2* mice were maintained as homozygous and confirmed by genotyping via qPCR, PCR and gel electrophoresis. E8.0, E8.5 and E9.5 *R26p-Fucci2* embryos were used for these studies. *Cdh5-Cre*^*ERT2*^,^43^ mice were a generous gift from Ralf Adams. *p27*^*fl/fl*^ (Jackson: 027328). *Cdh5-Cre*^*ERT2*^ *and p27*^*fl/fl*^ mice were crossed to create p27^fl/wt^ heterozygous and *Cdh5-Cre*^*ERT2*^ homozygous alleles and generate the *p27*^*fl/fl*^*;Cdh5-Cre*^*ERT2*^ mice. For *p27*EC^iKO^ mothers, tamoxifen (Sigma Cat# T5648) was resuspended in 10% ethanol and 90% corn oil (Sigma Cat# C8267) and was administered at 50mg/kg by intraperitoneal injection at E7.5 and E8.5, E8.5 and E9.5, or E9.5 and E10.5. E9.5, E10.5, and E11.5 *p27*EC^iKO^ embryos were used for these studies.

### METHOD DETAILS

#### Immunohistochemistry of embryos

Embryos were isolated and fixed in 4% paraformaldehyde (PFA) for one hour at 4°C, washed in 1X phosphate buffered saline (PBS) (Gibco: 10010023), cryopreserved using a 30% sucrose (Fisher Scientific: S5–3) + OCT (Sakura: 4583) solution, and frozen in OCT blocks. Embryos were then cryosectioned into 10μm sections onto slides (ThermoFisher Scientific: 22–037-246). For immunostaining, embryos were permeabilized in 0.05% Triton X-100 (Sigma: 9002–93-1) and 1X PBS, blocked for 1 h at room temperature using 0.01% Triton X-100 and 10% BSA (EMD Millipore: 126575–100GM) in 1X PBS, and then incubated overnight in blocking solution containing primary antibodies. Sections were then washed three times using 1XPBS +0.01% Triton X-100 and incubated with secondary antibodies in blocking solution for 1 h. Slides were washed three times with 1XPBS +0.01% Triton X-100, then mounted with glass coverslips and mounting media (Fluoromount-G, SouthernBiotech: 0100–01). Quantification of anti-pHH3 staining was conducted using ImageJ: EC nuclei containing pHH3 puncta were counted as positive. Quantification of cell death assay was conducted using automated structure identification in Imaris 10.2.0: using anti-CD31 to identify ECs, and DAPI to identify cell nuclei within CD31 stained area; cleaved caspase-3 puncta within those nuclei were quantified. All Antibodies used at 1:200 dilution.

#### Quantification of embryonic Fucci ECs In vivo

Images were quantified using FIJI,^47^ counting the number of ERG+ reporter-negative (early G1), ERG+ mCherry (late G1), and ERG+ mVenus (S/G2/M) ECs in arterial and venous vessels, identified via a combination of anti-DLL4, anti-EMCN, location, and morphology. The few identified G1/S ECs (ERG+ mCherry + mVenus) were counted as S/G2/M. The proportion of ECs in each population was calculated from total ERG+ cells per vessel. Plots were made and two-way ANOVA statistical tests were conducted using GraphPad Prism 10. Significance = *p < 0.05.*

#### Single cell digestion and fluorescence-activated cell sorting of embryonic ECs

Embryos were harvested at E8.0, E8.5, and E9.5, and the yolk sac of embryos was removed. For scRNAseq of wild-type embryos and bulk RNAseq of *R26p-Fucci2* embryos, embryos were pooled for digestion. For *p27*EC^iKO^ experiments, embryos were kept separate, and yolk sacs were genotyped for confirmation of the Cre recombinase and floxed gene. Embryos were disassociated into a single cell suspension in 1X HBSS (Gibco: 14025092) + 10% Fetal Bovine Serum (FBS) (Hyclone: SH30071.03) + 1% Collagenase II (determined by weight) (Worthington Biochemical: LS004176) at 37°C, with periodic dissociation using a p200 micropipette. E10.5 and 11.5 embryos were preliminarily mechanically disassociated using dissection scissors before enzymatic digestion. Digestions times: E8.0 = 5 min, E8.5 = 6 min, E9.5 = 7 min, E10.5 and E11.5 = 10 min. Cells were centrifuged at 300g at 4°C for 10 min and resuspended in 1X HBSS +10% FBS. Cells were filtered (Bel-Art products: H13680–0040) into a FACS tube and incubated with anti-CD31 BV421 and anti-CD45 per-CP antibodies for 45 min. CD31^+^ CD45^−^ ECs were sorted using a BD Biosciences FACSMelody and collected in 0.1% BSA-1X PBS for 10X Chromium v3 scRNAseq (processed by the University of Virginia Genome Analysis and Technology Core, RRID: SCR_018883) or lysis buffer for bulk RNAseq or RT-qPCR. Bulk RNAseq samples were submitted to NovoGene for low-input RNA sequencing.

#### Single cell library preparation and single cell RNA sequencing

Generation of indexed single-cell libraries was performed by the UVA School of Medicine Genome Analysis and Technology Core, RRID: SCR_018883. A 10X Genomics chromium controller platform was used with a Chromium Single Cell 3′ Library & Gel Bead Kit v3.1 reagent. 5,000 cells were targeted per sample. Quality control was performed using an Illumina Miseq with a nano 300Cycle kit (1.4 million reads/run), to estimate cell number. Based on this, approximately equal cell number was balanced per sample for deep sequencing using a NextSeq 2000, in conjunction with a P2–100 or P3–100 cycle kit. Binary base call files were demultiplexed and converted to fastq format using Illumina bcl2fastq2 software and then filtered feature matrices were generated using CellRanger v3.0.2 and used for downstream analysis.

#### qPCR for arterial and venous gene expression

RNA from lysed CD31^+^CD45^−^ ECs was extracted using commercial methods (Qiagen RNeasy Plus Micro: Qiagen, 74034). RNA was reverse transcribed (Fisher Scientific: 43–688-13) and qPCR (ThermoFisher: A25778) was performed. Gene expression was quantified using the delta-delta-Ct method for normalization to an internal control and comparison between samples. Significance = *p* < *0.05.*

#### Bioinformatic analysis of single cell RNA sequencing data

Single cell data was preprocessed in CellRanger v3.0.2 as described above and then filtered and processed using Seurat v5.1.0.^14,15^ All datasets were integrated after normalization using Seurat. Cells were identified using unbiased clustering, literature searches, and parallel bioinformatic analysis on published single cell datasets derived from developing embryo. ECs were selected based on *Pecam1, Kdr*, and *Cdh5* expression. Lineage projections were done using phateR,^18^ pseudotime was calculated using Monocle3.^19^ Module Scoring was conducted using Seurat: Arterial and Venous identity scores were determined using arterial-enriched genes: *Efnb2, Dll4, Hey1, Gja4, Gja5, Unc5b*, and venous-enriched genes: *Nr2f2, Nrp2, and Ephb4*, and *Flrt2*. Cell Cycle Progression and Arrest were determined using genes in Positive Regulation of Cell Cycle GO term (GO:0045787) and Cyclin Complex Inhibitors GO term (GO:0004861). Linear regression analysis was conducted in R. Significance = *p* < *0.05.*

#### Bioinformatic analysis of bulk RNA sequencing data

Data was quality controlled using FastQC (Andrews S. http://www.bioinformatics.babraham.ac.uk/projects/fastqc). Raw FASTQ sequencing output files were aligned to the mm10 UCSC mouse reference transcriptome using STAR aligner.^46^ Output was processed using DeSEQ2^45^ in R and timepoints were combined to find differences among different cell cycle states (early G1 vs. late G1 vs. S/G2/M). Differentially expressed genes were filtered by significance (*p* < 0.05) to obtain a list of differentially expressed genes within each cell cycle state. GO analysis on these genes was conducted using the GAGE package in R.^48^ GO-associated genes were subsequently used for Seurat Module Scoring. Significance = *p* < *0.05.*

#### Integration of bulk and single cell RNA sequencing data

Differentially expressed genes from separate Fucci cell cycle states were filtered by Log_2_ fold change into enriched (Log_2_ fold change >0) and downregulated (Log_2_ fold change <0) gene-sets per cell cycle state. Using Seurat’s Module Scoring functionality to score all individual cells within the scRNAseq dataset, we scored cells individually on genes that are significantly enriched and downregulated in each cell cycle state. To better define relationships between cell cycle and cell identity, enriched and downregulated module scores were scaled and combined to create a composite cell cycle enrichment score for each Fucci cell cycle state. Linear regression analysis was conducted in R.

#### 3D clearing and imaging of tissues

Embryos were dissected and prepared using an adaptation of a published protocol,^28^ outlined herein. Embryos were fixed for 1 h in 4% PFA, washed 3 times in DI water, and then delipidated in 50% Tetrahydrofuran (THF) + DI water overnight, and then 80% THF + DI water overnight again. Embryos were then washed in DI water 3 times for 3 min each. Embryos were blocked [in 2% BSA, 0.08% Triton X-100, 0.05% Sodium Azide (Sigma: S2002–5G) by weight in 1X PBS] (EZ Clear staining buffer) overnight. Blocked embryos were then immunostained in 1:100 Rat Anti-CD31 in EZ Clear staining buffer overnight at 4°C with mild agitation. Embryos were washed in 0.05% Triton X-100 in 1X PBS 3 times, 30 min each, and then incubated overnight in 1:200 secondary antibodies overnight at 4°C with agitation. Embryos were then washed with 0.05% Triton X-100 in 1X PBS 3 times, 30 min each wash, and mounted. To mount, 1mL needle-less insulin syringes were modified: the tip (needle end) was cleanly cut using a hot razor so the plunger could be inserted through this end. This was done so the agar cylinder could be extruded through the top of the syringe barrel to prevent rips and tears in the agar as this causes imaging artifacts. The syringe was reassembled in which the plunger was inserted through the modified end. A pipette was used to partially fill the syringe through the top with 1% molten agar dissolved in water. The syringe was left to cool for approximately 1 min (until the syringe was only warm to the touch) to prevent degradation of any immunostaining. Once the agar was slightly cooled, but still liquid, embryos were placed on top of the agar. To submerge the embryo within the agar, the plunger of the syringe was pulled down so the vacuum force would pull the embryo into the agar and to the desired height within the agar column. To ensure that embryos were correctly oriented, a small gauge needle or fine forceps were used to manipulate the embryo into an upright position, and syringes were placed upright to set. After agar was set, the agar cylinder was extruded by pushing the plunger and cut to size using a clean razorblade. This mounting protocol was based on and adapted from a previously published protocol.^49^ Mounted embryos were placed into EZ Clear solution and incubated at room temperature overnight with agitation. Representative imaging was performed using a Leica Thunder Widefield Microscope with embryos mounted on a glass slide and coverslip with a spacer between, immersed in mounting media. For 3D analysis of the vasculature, a 3i Light Sheet Imaging system or Zeiss LS7 system in UVA Advanced Microscopy Facility which is supported by the University of Virginia School of Medicine, Research Resource Identifiers (RRID): SCR_018736. For light sheet imaging, samples were immersed in EZ Clear solution with a layer of refractive Index (RI) matched Silicon + Mineral oil on top of the EZ Clear Solution to prevent evaporation and crystallization during imaging. Antibodies used at 1:100 dilution.

#### 3D image analysis of embryos

3D images were analyzed using Imaris 10.2.0. To create 3D volume of vasculature we used Imaris Machine learning. Foreground and background were determined manually and then iterated 2–3 times for optimal identification of signal. After 3D volume was created, the volume was masked, and then 3D Filament tools were used to create segments of 3D volume. Machine learning in this tool was used to determine optimal segments, and upon completion, Imaris statistics were run on 3D segments and 2D volume to determine total vessel area (vessel area), and mean segment diameter (vessel diameter). Significance = *p* < *0.05.*

### QUANTIFICATION AND STATISTICAL ANALYSIS

For single-cell RNA sequencing, differentially expressed genes were identified using Seurat^14,15^ utilizing a non-parametric Wilcox rank-sum test. For these experiments, E8.0, E9.5, E10.5 each contain two biological replicates (one litter per replicate): *n* = *2* combined litters per timepoint, total = 6 litters. Pseudotime was calculated using the Monocle3^19^ package which uses geodesic distance between cells using the PHATE 1 and 2 dimensions. Correlations were performed using simple linear regressions. Further statistical details can be found in the method details.

For Bulk RNA sequencing analysis, differentially expressed genes were identified using DESeq2,^45^ based on likelihood ratio tests and pairwise comparisons of samples. For bulk RNA sequencing of *R26p-Fucci2* embryos, three biological replicates for E8.0, E8.5, and E9.5 were collected (one litter per replicate). Biological samples *n* = *27* (3 timepoints, 3 cell cycle states, 3 replicates). For analysis, all like timepoints per cell cycle state were averaged in DESeq2. For example: E8.0, E8.5, and E9.5 early G1 timepoints were averaged for analyses. Final *n* = 9 per cell cycle state. Further statistical details can be found in the method details.

For imaging of *R26p-Fucci2 and p27*EC^iKO^ embryonic studies, each *n* represents quantifications from one embryo. Quantification and statistical details for all experiments can be found in the figure legends and method details.

## Supplementary Material

Supp Figures

Related to Fig 1

Related to Fig 2

Supp Movie S1

SUPPLEMENTAL INFORMATION

Supplemental information can be found online at https://doi.org/10.1016/j.celrep.2025.116368.

## Figures and Tables

**Table:** KEY RESOURCES TABLE

REAGENT or RESOURCE	SOURCE	IDENTIFIER

Antibodies		

Rabbit Anti-Erg	Abcam	Cat# ab92513; RRID: AB_2630401
Donkey Anti-Rabbit Alexa Fluor Plus 405	ThermoFisher	Cat# A48258; RRID: AB_2890547
Donkey Anti-Rabbit Alexa Fluor 405	Abcam	Cat# ab175664; RRID: AB_2313502
Rat Anti-CD31	BD	Cat# 550274; RRID: AB_393571
Donkey Anti-Goat Alexa Fluor 647	ThermoScientific	Cat# A-21447; RRID: AB_2535846
Donkey Anti-Rat Alexa Fluor 488	Invitrogen	Cat# A21208; RRID: AB_2535794
Donkey Anti-Rat Alexa Fluor 647	Abcam	Cat# ab150155; RRID: AB_2813835
Anti-phospho-histone H3 660	Invitrogen	Cat# 50–9124–41; RRID: AB_2574325
Anti-phospho-histone H3 488	ThermoFisher Scientific	Cat# 53–9124–82; RRID: AB_2784759

Chemicals, peptides, and recombinant proteins		

Tamoxifen	Sigma	Cat# T5648
Corn Oil	Sigma	Cat# C8267
PBS	Gibco	Cat# 10010023
Sucrose	Fisher Scientific	Cat# S5–3
OCT	Sakura	Cat# 4583
Super Frost Microscope Slides	ThermoFisher Scientific	Cat# 22–037–246
Triton X-100	Sigma	Cat# 9002–93–1
BSA	EMD Millipore	Cat# 126575–100GM
Fluoromount-G	SouthernBiotech	Cat# 0100–01
Fluoromount-G with DAPI	SouthernBiotech	Cat# 0100–20
HBSS	Gibco	Cat# 14025092
Collagenase II	Worthington Biochemical	Cat# LS004176
Pipette tip cell filters	Bel-Art products	Cat# H13680–0040
Sodium Azide	Sigma	Cat# S2002–5G
FBS	Hyclone	Cat# SH30071.03
EZ Clear solution	Hsu et al.,^28^ generously provided by Josh Wythe.	–

Critical commercial assays		

Qiagen RNeasy micro kit	Qiagen	Cat# 74104
Applied Biosystems High-Capacity cDNA Reverse Transcription Kit	Fisher Scientific	Cat# 43–688–13
PowerUp SYBR Green Master Mix for qPCR	ThermoFisher	Cat# A25778

Deposited data		

Single-cell RNA sequencing of E8.0, E8.5, and E9.5 embryonic ECs.	This study	NCBI GEO: GSE287112
Bulk RNA Sequencing of E8.0, E8.5, and E9.5 embryonic *Fucci2* ECs.	This study	NCBI GEO: GSE287115

Experimental models: Organisms/strains		

C57BL/6	Jackson Labs	Jax: 000664
*R26p-Fucci2*	Abe et al.^24^	Riken: CDB0203T
*Cdh5Cre* ^*ERT2*^	Sörenson et al.^43^	N/A
*p27^fl/fl^*	Jackson Labs	Jax: 027328 | 129S4/SvJae-Cdkn1b^tm2Mlf^/J

Oligonucleotides		

Primers for RTqPCR	This study	Table S3
Primers for Genotyping Mouse Strains	This study, Abe et al., ^24^ Jackson Labs Protocol: 27649, Wang et al. & Sorenson et al.^43,44^	Table S4

Software and algorithms		

Seurat	Hao et al.^10,11^	v5.1.0
phateR	Moon et al. ^18^	v1.0.7
Monocle3	Trapnell et al. ^19^	v1.3.7
DeSEQ2	Love et al.^45^	v1.42.1
STAR aligner	Dobin et al.^46^	v2.7.11b
fastQC	https://www.bioinformatics.babraham.ac.uk/projects/fastqc/	v0.11.5
Graphpad Prism	Graphpad	v10
FIJI	Schindelin et al. ^47^	v2.14.0
CellRanger	Illumina	V8.0.0
Adobe Illustrator	Adobe	N/A
Flowjo	BD Biosciences	v10.8.1
Imaris	Oxford Sciences	v10.2.0
R	R project	v4.3.3
RStudio	Posit	v2023.12.1 + 402

Other		

Leica DMi8 Lightning Confocal (SP8)	Leica	RRID:SCR_018169
Leica DMi8 Thunder Widefield	Leica	RRID:SCR_026034
FACS Melody	BD Biosciences	RRID:SCR_023209
3i Cleared Tissue Light Sheet System	3i	N/A
LS7 Light Sheet	Zeiss	RRID:SCR_024448
QuantStudio 6	Life Sciences	RRID:SCR_020239
Next Seq 2000	Illumina	RRID:SCR_023614
MiSeq	Illumina	RRID:SCR_016379

## Highlights

- Integrated transcriptomics of embryonic endothelial cells across cell cycle states
- Arterial and venous endothelial cells exhibit distinct cell cycle states before blood flow
- Endothelial cell cycle control is required for embryonic arterial-venous specification
