# Supplementary material for: Emergence of endothelial subtypes and role of cell cycle control in arterial-venous specification during embryonic vascular development
Source: Cell Rep. Author manuscript; Available in PMC 2025 Dec 8. (PMC12685131; doi:10.1016/j.celrep.2025.116368)
Supplement: Supp Figures [file NIHMS2116562-supplement-Supp_Figures.pdf]

Cell Reports, Volume *44*

Supplemental information

Emergence of endothelial subtypes and role of cell cycle control in arterial-venous specification during embryonic vascular development

Jordon W. Aragon, Elizabeth A. Nelson, Nicholas W. Chavkin, Madeline G. Jackson, Won Heo, Shelby R. Cain, Zaneta Markowska, Grace E. Bradecamp, Gael Genet, Aleksandra Ćwiek, and Karen K. Hirschi

Figure S1. Arterial and venous ECs are in transcriptionally distinct cell cycle states.

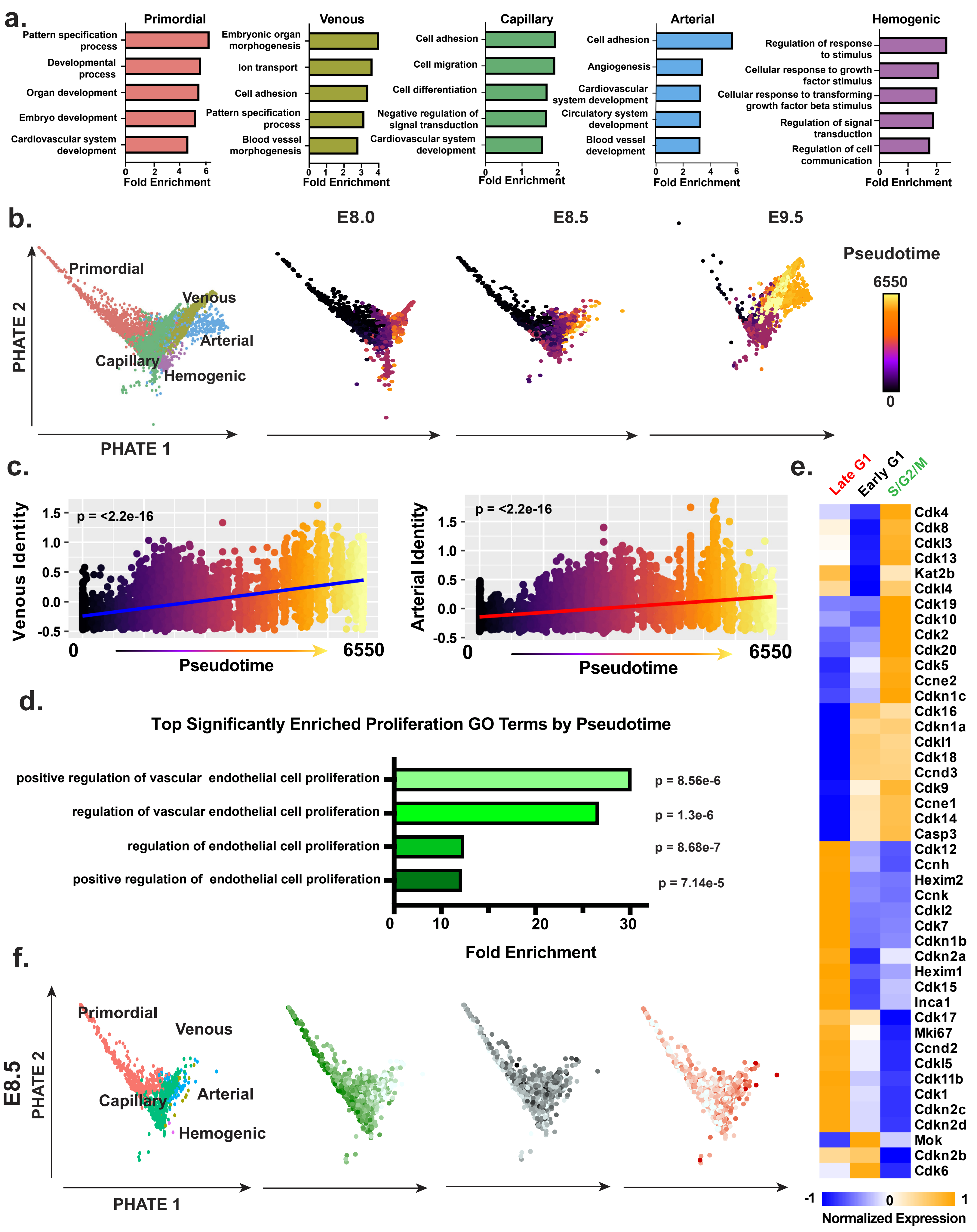

Figure S1: Arterial and venous ECs are in transcriptionally distinct cell cycle states. **(a)** GO analysis of Differentially expressed genes in each single-cell cluster. **(b)** PHATE lineage dimensionality reduction of all ECs, then separated by embryonic timepoint. ECs are colored based on Monocle3 pseudotime ordering. Low pseudotime = early time point, less specified, high pseudotime = later time point, more specified. **(c)** Simple linear regression analysis of arterial and venous identity scores, as a function of pseudotime. **(d)** GO term enrichment analysis of proliferation-related genes significantly enriched over pseudotime. **(e)** Heatmap of all significantly differentially expressed cell cycle genes per Fucci cell cycle state in developing endothelial cells. **(f)** Enrichment of differentially expressed genes in S/G2/M, early G1, and late G1 in individual E8.5 ECs plotted in PHATE dimensionality reduction plot.

Figure S2: ECs throughout E9.5 reside in distinct cell cycle states.

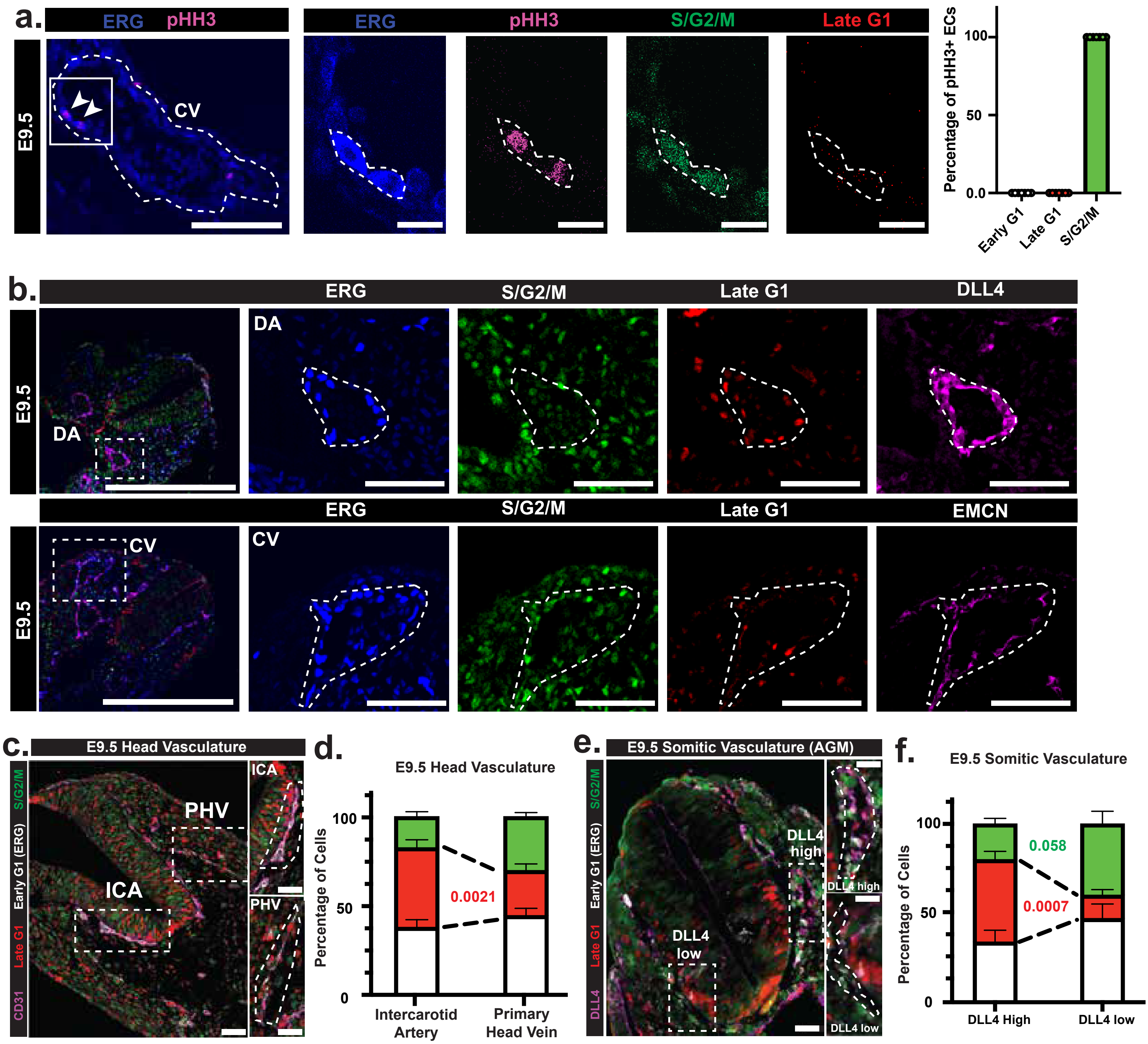

Figure S2: ECs throughout E9.5 embryos reside in distinct cell cycle states. **(a)** Representative confocal images and quantification of E9.5 *R26p-Fucci2* embryo section stained with anti-ERG and anti-phospho-histoneH3 antibodies indicating that proliferating cells only indicate S/G2/M ECs, arrows indicate ERG+ pHH3+ cells, scale bar = 100μm, magnified image of CV scale bar = 20μm. *n* = 7 embryos **(b)** Representative confocal images of E9.5 *R26p-Fucci2* embryo sections, scale bar =500μm. Magnified images of DA and cardinal veins (CV), scalebar = 100μm. **(c)** Representative confocal image of E9.5 *R26p-Fucci2* head section immunostained with EC nuclear marker anti-ERG1/2/3 and anti-CD31, to identify ECs in early G1 (ERG^{+}, reporter-negative), late G1 (ERG^{+} $mCherry^{+}$), and S/G2/M (ERG^{+} $mVenus^{+}$), scale bar = 170μm. Magnified images of intracarotid artery (ICA) and primary head vein (PHV), scale bars = 110μm. (**d**) Quantified cell cycle states in E9.5 ICA and PHV (SEM ±, 2way ANOVA, ICA and PHV *n* = 9 embryos). (**e**) Representative confocal image of E9.5 *R26p-Fucci2* somite section immunostained with immunostained with EC nuclear marker anti-ERG1/2/3 and anti-DLL4, to identify ECs in early G1 (ERG^{+}, reporter-negative), late G1 (ERG^{+} $mCherry^{+}$), and S/G2/M (ERG^{+} $mVenus^{+}$), scalebar = 100μm. Magnified image of $DLL4^{High}$ (arterial) and $DLL4^{Low}$ (venous), scalebar =60μm. (**f**) Quantified cell cycle states in E9.5 $DLL4^{High}$ and $DLL4^{Low}$ somitic ECs (SEM ±, 2way ANOVA, $DLL4^{High}$ and $DLL4^{Low}$ *n* = 8 embryos).

Figure S3. Whole-mount light sheet imaging of cleared p27ECiKO embryos and littermate controls.

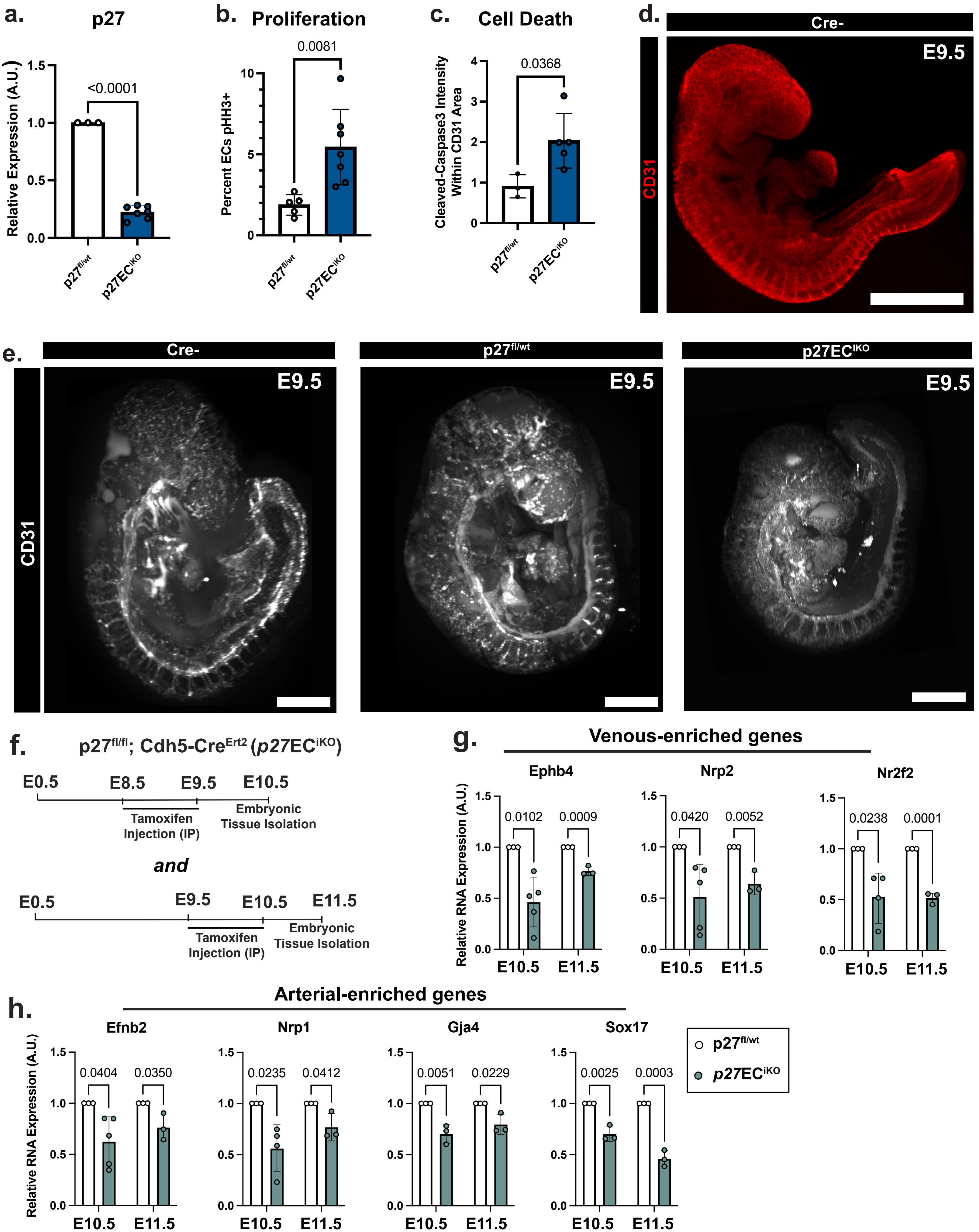

Figure S3. Whole-mount light sheet imaging of cleared p27EC^{iKO} embryos and littermate controls. **(a)** RT-qPCR expression of *Cdkn1b* in $CD3^{+}CD45^{-}$ ECs (*p27*$^{fl/wt}$ *n* = 3, *p27*EC^{iKO} n = 7 embryos). **(b)** Analysis of anti-pHH3 staining in $CD31^{+}$ ECs (*p27*$^{fl/wt}$ *n* = 4, *p27*EC^{iKO} n = 7). **(c)** Quantification of immunostaining with anti-CD31, DAPI and cleaved caspase-3 in sectioned E9.5 embryos (*p27*$^{fl/wt}$ *n* = 3 embryos, *p27*EC^{iKO} *n* = 5 embryos). **(d)** Representative widefield image of CDH5Cre^{ERT2} (-) E9.5 embryo immunostained with anti-CD31, scale bar = 500μm **(e)** Representative images of cleared, whole mount E9.5 CDH5Cre^{ERT2} (-), *p27*$^{fl/wt}$*,* and *p27*EC^{iKO} embryos imaged using light sheet microscopy, immunostained with anti-CD31, scale bar = 300μm. **(f)** Schematic of tamoxifen injections of *p27*EC^{iKO} at E8.5 and E9.5 or E9.5 and E10.5. **(g)** Venous- and **(h)** arterial- enriched gene expression in CD31+CD45- endothelial cells in E10.5 and E11.5 *p27*EC^{iKO} embryos vs littermate controls (*p27*$^{fl/wt}$ *n* = 3 embryos, *p27*EC^{iKO} *n* = 3-5 embryos). (SEM ±, all Unpaired *t*-tests, from multiple litters, *p27*EC^{iKO} paired with *p27*$^{fl/wt}$ littermate controls). Arbitrary Units (A.U.)

Table S3: Primer Sequences Related to RTQPCR – Related to STAR Methods

**Gene (Mouse)**	**5' – Forward – 3'**	**5' – Reverse - 3'**
Etv2	CCTGAAGTGGCTCTACAAGAGG	CCTGGAAAGGTACGTCTTCGTG
Hspg2	CATTCAGGTGGTCGTCCTCTCA	AGGTCAAGCGTCTGTCCTTCAG
Plvap	GTTGACTACGCGACGTGAGATG	AGCTGTTCCTGGCACTGCTTCT
Ephb4	GTGCTGGACTACGAGGTCAAGT	TACCTGGACCAGATAGCTGGCT
Nrp2	GGTGAAGATTGGATGGTCTACCG	TGAACCGAGTCAGCAGTGGCAT
Nr2f2	CGGCGAGTATACTG CCTCAAG	CTGGCTCCTAACGTACTCTTCC
Efnb2	CCAACAAGACGTCCAGAGCTAG	CCACTTCGGAACCCAGGAGATT
Nrp1	CGGAGGAATGTTCTGTCGCTATG	GGATAGAACGCCTGAAGAGGAG
Gja4	AACGGTGCTCTTCATCTTCCGC	GGTCATAGCAGACGTTGGTGCA
Sox17	GCCGATGAACGCCTTTATGGTG	TCTCTGCCAAGGTCAACGCCTT
Cdkn1b (p27)	AGCAGTGTCCAGGGATGAGGAA	TTCTTGGGCGTCTGCTCCACAG

Table S4: Primer Sequences Related to Mouse Strain Genotyping – Related to STAR Methods

**Transgene**	**5' – Forward – 3'**	**5' – Reverse - 3'**
Cdh5:Cre^{ERT2}	CAGATCAGCTCCTCCACGAA	TGG TGGGCAGGTAGCATGTT
Cdh5:Cre^{ERT2} Reverse 2		CATTGCTGTCACTTGGTCGT
R26p-Fucci2 (mVenus)	ATGGTGAGCAAGGGCGAGGAG	CTTGTACAGCTCGTCCATGCC
P27fl/fl	TGGATGTTTATAGGGGAAATGG	CTCCCATCCAATTCGACAAC
qPCR-Fucci2 (mVenus)	AAGGACGACGGCAACTACAA	AAGTTGGCCTTGATGCCGTT
